# The Novel Compound Heterozygous Mutations in the AGL Gene in a Chinese Family With Adult Late-Onset Glycogen Storage Disease Type IIIa

**DOI:** 10.3389/fneur.2020.554012

**Published:** 2020-11-19

**Authors:** Qianqian Qu, Qi Qian, Jiejing Shi, Haiyan Liu, Yan Zhang, Wenhao Cui, Ping Chen, Haidong Lv

**Affiliations:** ^1^Department of Neurology, The People's Hospital of Jiaozuo City, Jiaozuo, China; ^2^Graduate School of Xinxiang Medical University, Xinxiang, China

**Keywords:** glycogen storage disease type III, glycogen debrancher enzyme, gene mutation, skeletal muscle MRI, AGL gene

## Abstract

**Objective:** To investigate the clinical features, skeletal muscle imaging, and muscle pathological characteristics of late-onset GSD IIIa caused by mutation of the AGL gene in adults.

**Methods:** The clinical data, skeletal muscle imaging, pathological data, and gene test results of a family with late-onset GSD IIIa in adulthood were collected in detail in November 2019.

**Results:** The proband is a 40-years-old male, who was admitted into our hospital due to a 2-years history of limb weakness. The proband was diagnosed with the following syndrome: he had a 15-years history of elevated muscle enzymes; the cranial nerve examinations showed no abnormal findings; the muscle tension in both upper and lower limbs was low, and tendon reflexes were absent; the proband's muscle strength was 5 in the proximal muscles and 4 in the distal muscles of the upper limbs, with 3 in the proximal muscles and 4 in the distal muscles of the lower limbs; Magnetic Resonance Imaging (MRI) revealed abnormally high signal intensity changes in the posterior thigh muscle group, and the posterior-medial calf muscle group; and vacuoles were evident in some muscle fibers biopsied from the gastrocnemius muscle. Periodic acid-Schiff staining stained the cytoplasm of muscle fibers a dark red color. The proband's older brother exhibited the same clinical features. DNA analysis identified mutations in the AGL gene in the proband, his older brother, and parents. The proband and his older brother both carried two compound heterozygous mutations, c.866G>A and c.2855_2856insT. Pedigree analysis demonstrated that c.866G>A and c.2855_2856insT mutations had been inherited from the mother and father, respectively.

**Conclusion:** Late-onset GSD IIIa in adults is clinically characterized by muscle weakness, muscle atrophy, and mainly occurred in the posterior thigh muscle group. We also identified two novel compound heterozygous mutations (c.866G> A and c.2855_2856insT) in the AGL gene.

## Introduction

Glycogen storage disease type III (GSD III) is a rare autosomal recessive genetic disorder caused by the deficiency of the glycogen debranching enzyme. Due to this deficiency, the glycogen debranching enzyme cannot break down the chains of glycogen normally. This causes glycogen accumulation in the liver, skeletal muscle, and myocardium, resulting in liver enlargement, fasting hypoglycemia, delayed normal growth, and progressive muscle weakness (1). GSD IIIa is mostly diagnosed in infants and during early childhood; late-onset GSD IIIa in middle age is relatively rare (2). This study reported a patient aged 40 years who was diagnosed as GSD IIIa by muscle biopsy. The clinical data, skeletal muscle imaging, pathological data, and genetic testing results of the family were collected and analyzed in detail. Genetic tests were performed for the proband and his family members with written informed consent. The results showed that there were two compound heterozygous mutations in the patient-related gene AGL, and two novel mutations (c.866G>A and c.2855_2856insT) in the AGL gene. Our findings provide evidence of a wider spectrum of mutations in patients with GSD IIIa in the Chinese population.

## Methods and Materials

### Collection of Clinical Information

The proband is a 40-years-old male who was admitted into our hospital on November 21st, 2019. After a muscle biopsy, the clinical diagnosis was considered as glycogen storage disease. Four family members provided relevant medical history and completed genetic testing after signing informed consent forms.

This study was approved by the Ethics Committee of Jiaozuo People's Hospital and guardians of the patient signed the written informed consent for the research.

### Neuro-Electrophysiological Examination

Concentric needle electromyography, along with sensory and motor nerve conduction velocities, was measured by an electromyography evoked potentiometer (Photoelectric MEB-9200K, Japan).

### Muscle MRI

Magnetic resonance imaging (MRI) was performed on both lower limbs using a 3.0 Tesla MR scanner (GE Healthcare, Milwaukee, USA); all images were acquired in an axial plane. Sequences from MRI scans included T1-weighted (T1WI), T2-weighted (T2WI), and IDEAL sequences.

#### Pathological Examination of the Muscle

The patient underwent an open muscle biopsy in the right gastrocnemius muscle after local anesthesia. The muscle specimens were immediately frozen in liquid nitrogen-cooled isopentane. Then cryosections were prepared for pathology evaluation. Frozen muscle sections were stained with hematoxylin eosin (H&E) and modified Gomori trichrome (abbreviated mGT), oil red O (ORO), and periodic acid-Schiff (PAS). Sections were also stained for cytochrome C oxidase (COX), adenosine triphosphatase (ATPase), and reduced nicotinamide adenine dinucleotide tetrazolium reductase (NADH-TR). Stained sections were analyzed by light microscopy.

### Genetic Analysis

Peripheral blood samples (4–6 ml) from the patient, his brother, and his parents were sent to Running Gene, Inc. (Beijing, China), for targeted next-generation sequencing analysis using the neurological disorder panel. In brief, genomic DNA was extracted from peripheral blood leukocytes and sequenced by Illumina NovaSeq (Illumina, USA). Raw data were assessed by Illumina Sequence Control Software (SCS), and then bioinformatic analysis was performed to identify the mutations in AGL gene.

## Results

### Clinical Features

The proband, a 40-years-old male, was admitted to our hospital with the chief complaint of “muscle enzymes elevated for 15 years, limb weakness for 2 years, and aggravation^1^ for 1 year.” Laboratory exams performed 15 years ago showed that he had abnormal liver function, with significantly elevated levels of transaminases and muscle enzymes. However, he did not have hepatitis, heart disease, or symptoms of muscle weakness. The proband could perform normal physical labor. Two years prior to the current diagnosis, related clinical symptoms appeared, such as progressive weakness in both lower limbs and difficulty in walking upstairs and standing up after squatting. He could no longer perform heavy physical labor. One year prior to the current diagnosis, the weakness in both lower limbs had worsened. He required stair handrails when going upstairs and had difficulty standing up after squatting; muscle weakness in the hands increased. Six months prior to the current diagnosis, muscle atrophy appeared in both hands and lower limbs. The weakness in his limbs affected his daily life significantly. Clinical tests performed in the local hospital showed elevated levels of myocardial enzymes, although no evidence of abnormalities was found through coronary angiography. However, electromyography performed 1 month prior to admission to our hospital showed evidence of myogenic lesions. A muscle biopsy was recommended for further diagnosis. Subsequently, the patient was transferred to our hospital.

On admission, routine physical examination showed that the proband had developed normally. He is 170 cm in height, and his weight is 68 kg, with normal intelligence. Neurological examination showed no abnormal findings in the cranial nerves. He had good strength in his neck when lifting his head. Muscle tension in the upper limbs was slightly low, and tendon reflexes were absent. His upper limb strength was graded as 5 and 4 in the proximal and distal muscles, respectively. Muscle tension in both lower limbs was low, and tendon reflexes were absent. Muscle strength in the proximal lower limbs was graded as 3, the distal muscle strength of foot dorsiflexion was graded as 4, and the muscle strength of foot plantarflexion was graded as 3. The patient was unable to walk on the sole forefoot. Deep and superficial sensation was normal, and so was coordination. There were no pathological symptoms in the upper and lower limbs. Significant muscle atrophy was observed in the bilateral infraspinatus, pectoralis major, thenar, hypothenar, interosseous, and gastrocnemius muscles. Mild atrophy was observed in the quadriceps and the tibialis anterior muscle. No tenderness was observed in the limb muscles. The bilateral foot arches were normal.

The proband's parents were in a non-consanguineous marriage and are both healthy. The proband has one older sister and one older brother. His older sister is healthy, and his older brother is 163 cm tall and weighs 55 kg, with a curved backbone. His older brother had the same clinical symptoms of muscle weakness. Neurological examination showed no abnormal findings in the cranial nerves, with normal intelligence. His muscle strength was scored 4 in the upper limbs and 3–4 in the lower limbs; The symmetry of the tendon reflex of both upper and lower extremities were absent; both right and left pathological signs were negative; both right and left depth and shallow sensation were normal; muscle atrophy is present in upper and lower limbs. As a result, he could not walk normally and is unable to perform normal physical labor at home.

#### Neuroelectrophysiological Examinations

Results from needle electromyography revealed abnormal spontaneous potentials in some muscles, a reduction in the amplitude of motor unit action potentials in the muscles and an increase in the percentage of polyphasic waves in the motor unit potentials. These results indicated myogenic lesions. Nerve conduction measurements showed that conduction speeds in the left and right nervus medianus were 1.2 and 0.9 mv, respectively. These values are significantly lower than the normal range (≥5.0 mv). Motor conduction speed in the right was 43.9 m/s, significantly lower than the normal range (≥50 m/s).

#### Laboratory Examinations

Cardiac color Doppler ultrasonography showed thickening of the interventricular septum and the left ventricular posterior wall; echoes were uneven, and the left ventricular ejection fraction was 58%, indicating that the patient had non-obstructive hypertrophic cardiomyopathy. Electrocardiography showed left ventricular hypertrophy and low-to-flat T waves. Color Doppler ultrasonography showed that the size and morphology of the liver were both normal, that the echo of the liver parenchyma was coarse, and that the portal vein was not enlarged, suggesting that the liver was only mildly involved.

Laboratory test results were as follows: creatine kinase (CK, 5121 U/L); CK MB isoenzyme (CKMB, 405 U/L); lactate dehydrogenase (LDH, 1563 U/L); alanine transaminase (ALT, 159 U/L); aspartate aminotransferase (AST, 213 U/L); LAC (1.76 mmol/L); and Glu (4.6 mmol/L).

#### MRI Findings in the Skeletal Muscle

**Figure 1:**
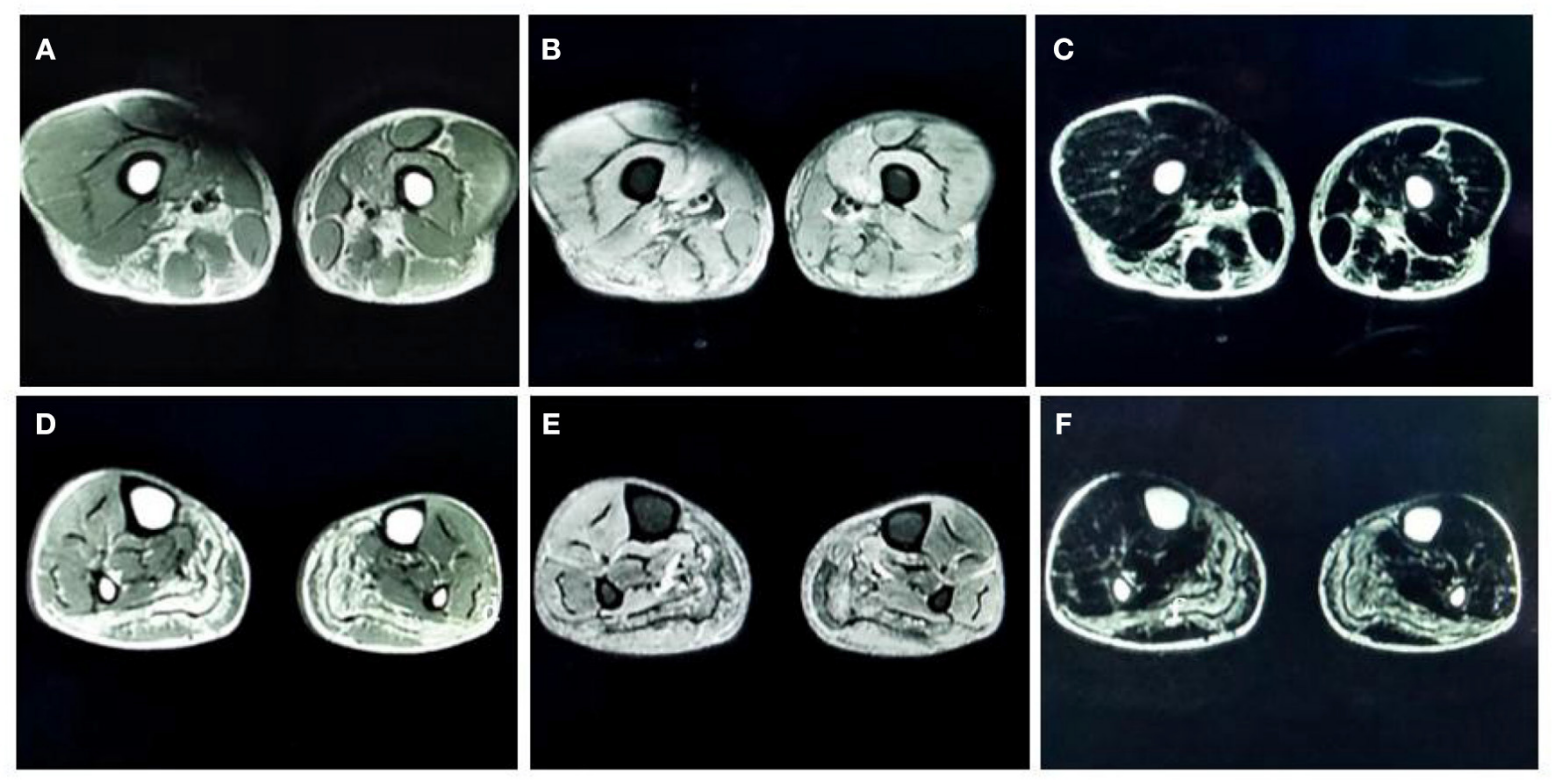
**(A)** T1-weighted (T1-WI) magnetic resonance imaging (MRI) of both thighs showed high signal intensities in the adductor magnus, biceps femoris, semitendinosus, and semimembranosus muscles. **(B)** Fat-suppressed T2-weighted (T2WI) images of both thighs showed high signal intensities in the vastus medialis and intermedius. **(C)** Water-suppressed T2WI MRI images of both thighs showed high signal intensities in the adductor magnus, biceps femoris, semitendinosus, and semimembranosus muscles. **(D)** T1-WI images of both lower legs showed diffuse high signal intensities in the medial and lateral heads of the gastrocnemius muscle and part of the soleus muscle. **(E)** Fat-suppressed T2WI images of both lower legs showed slightly higher signal intensities in the gastrocnemius muscle and part of the soleus muscles. **(F)** Water-suppressed T2WI MRI images of both lower legs showed high signal intensities in the gastrocnemius muscle and part of the soleus muscles.

MRI of the muscles in both lower legs showed abnormally high signal intensity in the posterior compartment muscles of thigh and the posterior-medial compartment of the calf (Figure 1).

#### Pathological Changes in the Skeletal Muscle

**Figure 2:**
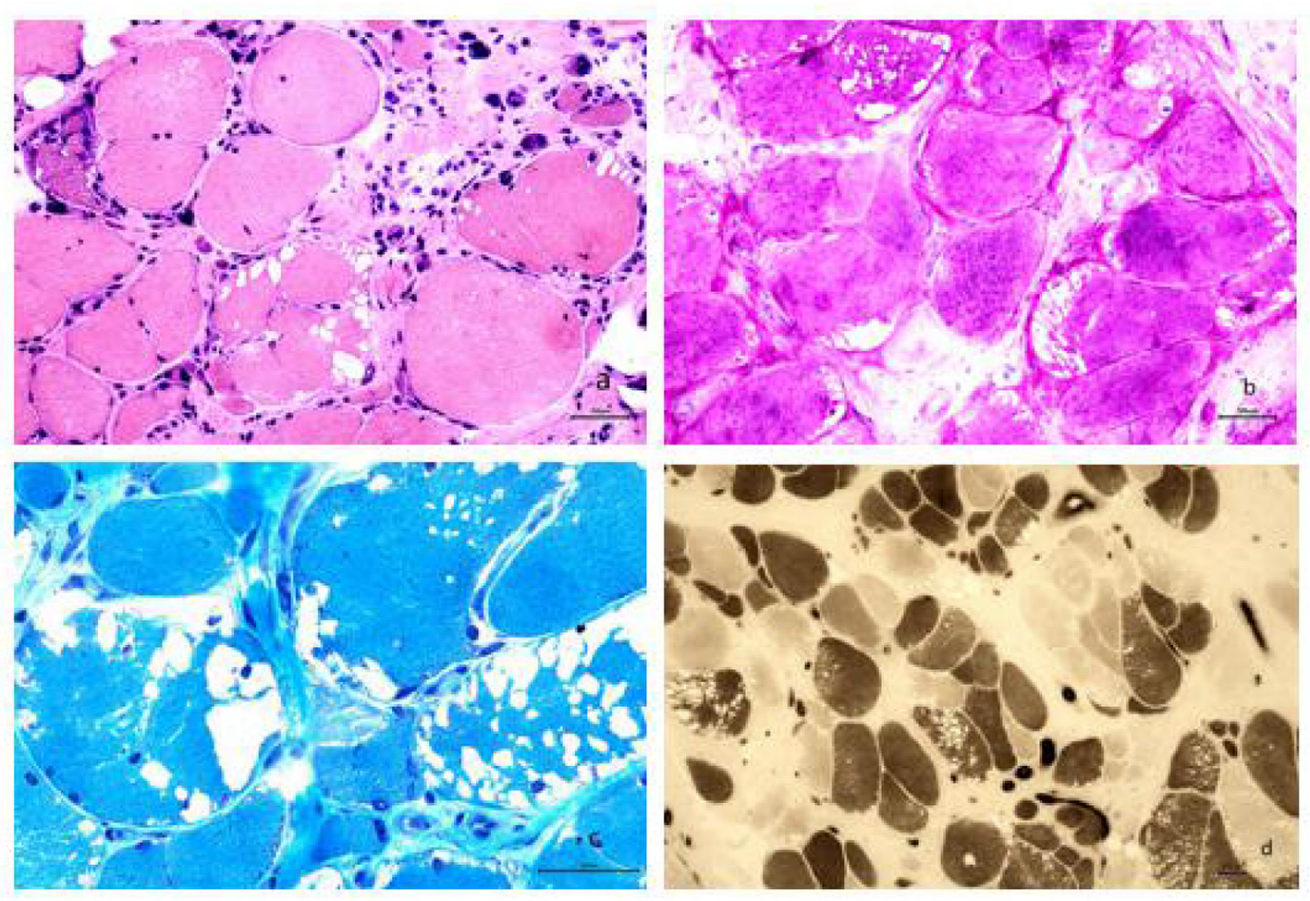
**(a)** Analysis revealed muscle fibers of different sizes; additionally, many small and rounded atrophic fibers and compensatory hypertrophic muscle fibers were found. We also observed a large number of vacuoles in some muscle fibers, pyknosis of the nuclei, and splitting of the muscle fibers (HE staining, × 200). **(b)** The cytoplasm of most muscle fibers stained red, indicating the accumulation of glycogen. The vacuoles evident in some muscle fibers were considered to be caused by the loss of glycogen and were mainly localized under the sarcolemma of the muscle fibers (PAS staining, × 200). **(c)** Modified Gomori trichrome staining revealed a large number of vacuoles beneath the sarcolemma of the muscle fibers; however, no rimmed vacuoles or ragged-red fibers were seen (MGT staining, × 400). **(d)** ATPase staining further demonstrated that the distribution of type I and II muscle fibers occurred in a mosaic pattern, that muscle fibers were affected on both sides, and that there was no apparent grouping of fibers by specific type (ATPase staining, × 100).

A biopsy from the right gastrocnemius muscle showed that the muscle fibers were of different sizes with many small and rounded atrophic fibers and compensatory hypertrophic muscle fibers. We also observed a large number of vacuoles in some muscle fibers, pyknosis of the nuclei, and splitting of the muscle fibers. PAS staining resulted in the cytoplasm of muscle fibers staining a dark red color. MGT staining failed to show any evidence of rimmed vacuoles and ragged-red fibers. ATPase staining demonstrated that the distribution of type I and II muscle fibers was normal, although muscle fibers were affected on both sides (Figure 2).

#### Genetic Testing

**Figure 3:**
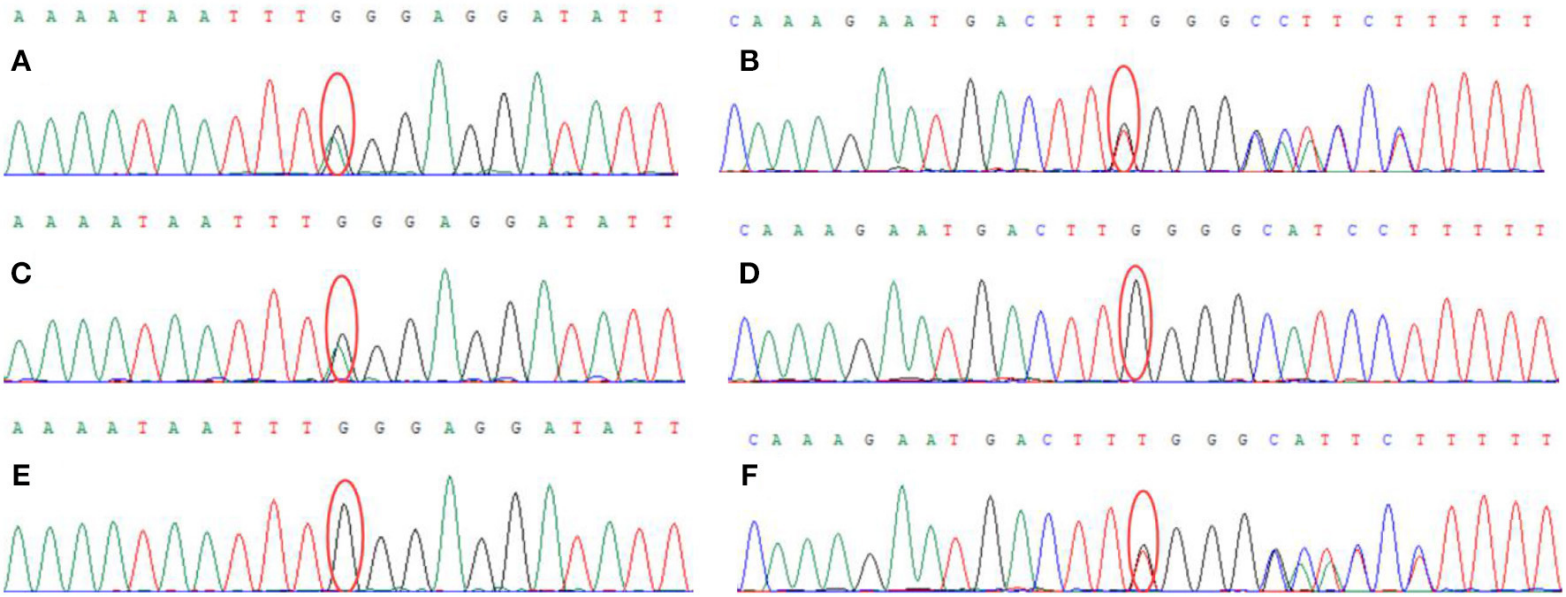
Genetic analysis showed that the proband carried two heterozygous mutations: **(A)** c.866G > A (chr1:100336333), **(B)** c.2855_2856insT (chr1: 100356816). The proband's mother carried the c.866G > A (chr1:100336333) mutation **(C)** but did not carry the c.2855_2856insT (chr1: 100356816) mutation **(D)**. The proband's father did not carry the c.866G>A (chr1:100336333) mutation **(E)** but did carry the c.2855_2856insT (chr1: 100356816) mutation **(F)**.

Genetic analysis revealed two heterozygous mutations (c.866G>A and c. 2855_2856insT) in the AGL gene in both the proband and his older brother. The c.866G>A mutation resulted in the substitution of guanine for adenine and was a functional mutation that resulted in a significant change in the amino acid sequence (p.W289^*^; tryptophan > termination). The c.2855_2856insT mutation caused an insertion, resulting in an amino acid change (p.L952Ffs^*^7; a frameshift mutation that generates a termination codon at position 7) (Figure 3). These two heterozygous mutations had been inherited from the mother and father, respectively.

## Discussion

The case of GSD III was first reported in 1952 by Cori (3) and then by Forbes (4). Their studies revealed that the patient had suffered from the extensive accumulation of glycogen with short outer chains in the liver and muscle tissues. Enzymatic analysis of affected tissues has shown that GSD type III has four subtypes: IIIa, IIIb, IIIc, and IIId (5, 6). GSD IIIa is the most common subtype, accounting for ~80% of all cases of GSD III. GSD IIIa affects both the liver and muscle. GSD IIIb accounts for ~15% of all cases of GSD III, only affecting the liver. GSDIIIc and d rarely occur in humans. The onset of GSD IIIa mostly occurs in childhood and is often associated with recurrent fasting hypoglycemia and liver enlargement. With aging, the main clinical manifestation is myopathic symptoms (6).

In the present case, the proband underwent a physical examination at the age of 25 years and was shown with increased levels of liver enzymes, without liver enlargement. The patient did not experience any hypoglycemic episodes. He started to show symptoms of muscle weakness at the age of 38 years and was diagnosed with cardiovascular disease in a local hospital. Due to increasing muscle weakness in the past 2 years, the patient came to our hospital for further diagnosis and was diagnosed as GSD IIIa *via* pathology evaluation of a muscle biopsy and genetic testing. We consider this patient as having late-onset GSD IIIa in adulthood. This patient has specific features of muscle weakness in the upper and lower limbs: the distal upper limb muscles were more severely affected than the proximal muscles, and the proximal muscles of the lower limbs were more severely affected than the distal muscles. A physical examination revealed different degrees of muscle atrophy in the upper and lower limbs. Color Doppler ultrasound showed that the size of the proband's liver was normal, with no enlargement of the portal vein, which suggested that the liver was only mildly involved. These characteristics support the diagnosis of GSD IIIa, characterized by obvious muscle damage.

Previous literature reported that GSD IIIa has detrimental effects on cardiac function (5, 6). Although the proband had no clinical symptoms of heart disease, electrocardiography revealed left ventricular hypertrophy and flat T waves. Cardiac color Doppler ultrasonography further revealed thickening of the interventricular septum and the left ventricular posterior wall, indicating that the heart had been affected.

There is no consensus as to whether GSD IIIa is associated with peripheral neuropathy (6–8). In the present study, the amplitude of motor conduction in the bilateral median nerve and the motor conduction velocity in the right median nerve are reduced, but the nerve conduction tests in the other motor nerves are normal. We consider that the decrease in motor conduction speed in nervus medianus are probably related to muscle atrophy in the distal upper limbs, but not lesions in the axon of the peripheral nerve. Furthermore, electromyography showed myopathic changes, and a muscle biopsy showed no neurogenic damage. Thus, we consider that there is no pathological impairment of the peripheral nerves in this case.

Very few studies have investigated muscle MRI findings in patients with GSD IIIa. Two previous studies demonstrated edema and fat degeneration in the muscle fibers located in the posterior compartment of the lower limbs in patients with GSD IIIa (9, 10). Our case also found the similar results. In our case, we acquired abnormally high signal intensity in the adductor magnus, biceps femoris, semitendinosus, and the semimembranosus muscles in T1WI images. These findings indicated fat degeneration in those muscles. We found high signal intensities in the vastus medialis and intermedius in fat-suppressed T2WI images, suggesting that the thigh muscles were suffering from local edema. T1WI images of both lower legs showed diffuse high signal intensities in the medial and lateral heads of the gastrocnemius muscle and part of the soleus muscle, thus suggesting fat degeneration in the calf muscles. Furthermore, fat-suppressed T2WI images acquired from both legs showed slightly higher signal intensities in the gastrocnemius muscle and part of the soleus muscles, thus suggesting mild edema in the calf muscles. Collectively, consistent with previous studies (9, 10), these MRI findings indicate that patients with GSD IIIa may be mainly affected in the posterior compartment muscles of both proximal and distal lower extremities.

Skeletal muscle biopsies play a significant role in the diagnosis of GSD III. The typical pathological feature of GSD IIIa is the presence of a large number of vacuoles in the muscle fibers (5, 7), and the number of vacuoles varies greatly between different muscle bundles and muscle fibers. However, patients with GSD IIIa do not exhibit basophilic granules around the vacuoles. This is the feature for GSD IIIa, different for GSD II (5). Results from Laforêt et al. indicated that there was no correlation between clinical severity and degree of vacuolar myopathy based on 30 patients with GSD III (11). In the present study, PAS staining revealed that the cytoplasm of muscle fibers stained dark red, indicating that a large amount of glycogen had accumulated in the muscle fibers. Our analysis also revealed that the patient has different sizes of muscle fibers. We observed a large number of small and rounded atrophic fibers along with compensatory hypertrophic muscle fibers. We also observed the internal migration of nuclei and the splitting of muscle fibers. These results indicated that the late-onset of GSD IIIa in adulthood was associated with chronic myopathies. Unfortunately, electron microscopy is not available in our hospital for running additional relevant tests. We will try to include this in future studies when the equipment is available.

The AGL gene, located on the short arm of chromosome 1 (1p21), consisting of 35 exons and is 85 kb in length (12, 13). The Human Gene Mutation Database (HGMD; http://www.hgmd.cf.ac.uk) currently lists more than 259 mutations in the coding region of the AGL gene (14), including a range of homozygous or compound heterozygous mutations. Significant heterogeneity is also known to exist between the AGL gene mutations when compared between different ethnic groups (14). The most common AGL mutations are missense mutation, splice site mutations, and small deletion and insertion mutations. In our case, genetic analysis of DNA extracted from the blood of the proband identified two heterozygous mutations (c.866G>A and c.2855_2856insT) in the AGL gene. The patient carried two compound-heterozygous variants of AGL (NG_012865, NM_000642.2, and NP_000633.2). The maternal missense mutation c. 866G>A in exon 7 introduces a premature terminator and results in a truncating protein (p.W289^*^), leading to the loss of protein function (PVS1). The mutation is absent from controls (1000 Genomes, ExAC, gnomAD, and CNGB) (PM2). Multiple computational software programs predict that the mutation is probably a deleterious mutation (PP3). MutationTaster2 and MutPred-LOF programs predict that the mutation is disease causing, which might affect normal functions of the protein (15, 16). Therefore, mutation c.866G>A is considered a pathogenic mutation. On the other hand, the AGL mutation with a paternal origin c.2855_2856insT is a 1 bp intragenic insertion encompassed in exon 22, which leads to a shift in the open reading frame and a premature truncation of the protein (p.L952Ffs^*^), resulting in the loss of protein function (PVS1). The mutation is absent from controls (1000 Genomes, ExAC, gnomAD, and CNGB) (PM2) and is in trans with the pathogenic variant c.866G>A (PM3). It is also predicted to be a deleterious mutation by software programs MutationTaster2 and MutPred-LOF (PP3). Thus, mutation c.2855_2856insT is classified as pathogenic mutation of AGL according to the standard of ACMG (17). These two mutations (c.866G>A and c.2855_2856insT) are novel and are not listed in either the human gene database (http://www.hgmd.org/) or the HGMD. The patient was diagnosed with glycogen storage disorder as the results of a muscle biopsy, which were consistent with that of next-generation sequencing. Although we have also identified eight variants of unknown clinical significance (VUS) in this patient, all VUS variants were excluded due to their mismatches on symptoms between the patient and these VUS-caused diseases.

GSD IIIa is mostly treated by diet adjustments and exercise therapy. Enzyme replacement therapy has yet to be applied during the treatment of GSD IIIa (7, 8, 18). Over the last few years, researchers have made significant progress in the field of gene therapy (18, 19). These new treatments have now entered the clinical trial stage. It is expected that gene therapy may be more effective in the future for the treatment with GSD IIIa.

In summary, we analyzed a Chinese family with GSD IIIa using a range of clinical and genetic testing. Our results showed that GSD IIIa in adulthood is clinically characterized by muscle weakness, muscle atrophy, and elevated levels of muscle enzymes. MRI tests showed that patients with GSD IIIa were predominantly affected in the posterior compartment muscles of both proximal and distal lower extremities. We identified two novel mutations (c.866G>A and c.2855_2856insT) in the AGL gene. To the best of our knowledge, these two compound heterozygous AGL mutations have not been reported previously. Our findings provide evidence of a wider spectrum of mutations in patients with GSD IIIa in the Chinese population.

## Ethics Statement

Written informed consent was obtained from the individual for the publication of this case report, including any potentially identifiable images or data included in this article.

## Author Contributions

QQu is responsible for writing. QQi and JS are responsible for data collection. WC and PC are responsible for making important revisions to papers. HLi and YZ are responsible for data analysis. HLv is responsible for overall study design and supervision. All authors contributed to the article and approved the submitted version.

## Conflict of Interest

The authors declare that the research was conducted in the absence of any commercial or financial relationships that could be construed as a potential conflict of interest.

## Footnotes

^1^Worsening of clinical symptoms.
